# Supplementary material for: Eley–Rideal model of heterogeneous catalytic carbamate formation based on CO2–MEA absorptions with CaCO3, MgCO3 and BaCO3
Source: R Soc Open Sci. 2019 May 1;6(5):190311. doi: 10.1098/rsos.190311 (PMC6549977; doi:10.1098/rsos.190311)
Supplement: Supplementary materials A the rate equation development [file rsos190311supp1.docx]

**Supplementary Materials (A)**

**The development of Elementary reaction steps from Eley-Rideal models**

# **The Eley – Rideal model was developed based on the reactions of CO_2_ + MeOH.**

Fig. S1. Elementary mechanistic steps proposed for the formation of dimethyl carbonate (DMC) from CO_2_ and methanol by an Eley–Rideal mechanism

Ref 25: V. Eta, P. Maki-Arvela, J. Warna, T. Salmi, J.P. Mikkola, D.Y. Murzin, Appl. Catal. A 404 (2011) 39–46.

This research developed Elementary reaction steps based on similar reactions, with CO_2_ and Eley-Rideal models. The other chemicals (b) adsorbed onto solid surface, and CO_2_ react with it directly.


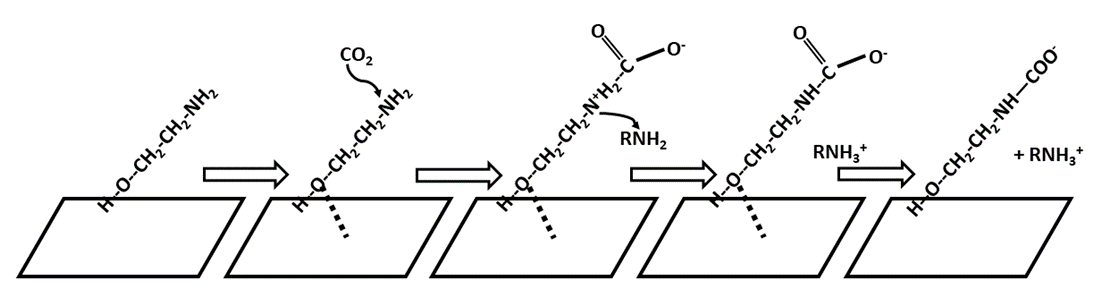


**The Elementary steps based on Figure 1**

| **B1** | \| 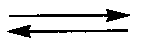RNH_2_ + (*) RNH_2(*)_ \| \| --- \| | *k_1_, k-_1_* |
| --- | --- | --- | --- |
| **B2** | \| 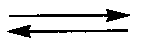CO_2_ _(g)_ + RNH_2(*)_  RNH_2_^+^-COO^-^_(*) (Zwitterion)_ \| \| --- \| | *k_2_, k-_2_* |
| **B3** | H_2_O + RNH_2_^+^-COO^-^ _(*)_🡪RNH-COO^-^ _(*)(Carbamate)_ + H_3_O^+^ | *k_3_, k-_3_* |
| **B4** | \| 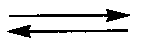RNH-COO^-^ _(*)_ RNH-COO^-^ _(Carbamate)_ + (*) \| \| --- \| | *k_4_, k-_4_* |

**The Elementary steps for Eley - Rideal model with Apparent Rate Law Derivation**

# **The Catalytic carbamate formation from Eley - Rideal model:**


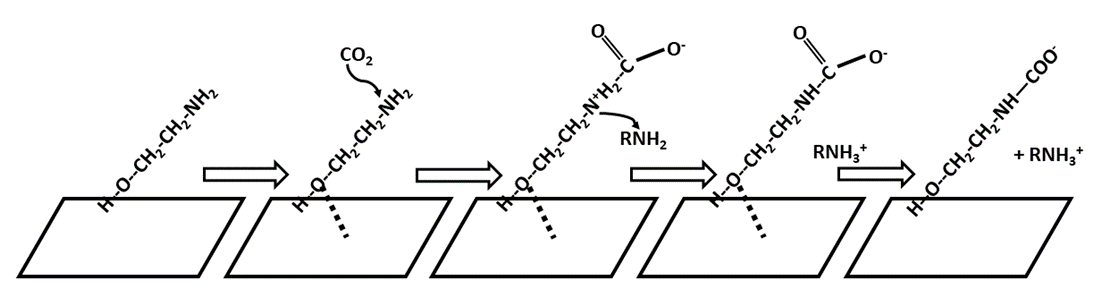


**The derivation of Apparent rate law was similar from Ref.[26]**

**The Elementary steps based on Figure 1**

| **B1** | \| 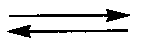RNH_2_ + (*) RNH_2(*)_ \| \| --- \| | *k_1_, k-_1_* |
| --- | --- | --- | --- |
| **B2** | \| 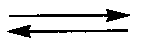CO_2_ _(g)_ + RNH_2(*)_  RNH_2_^+^-COO^-^_(*) (Zwitterion)_ \| \| --- \| | *k_2_, k-_2_* |
| **B3** | H_2_O + RNH_2_^+^-COO^-^ _(*)_🡪RNH-COO^-^ _(*) (Carbamate)_ + H_3_O^+^ | *k_3_, k-_3_* |
| **B4** | \| 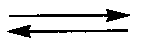RNH-COO^-^ _(*)_ RNH-COO^-^ _(Carbamate)_ + (*) \| \| --- \| | *k_4_, k-_4_* |

Abbreviations: A = Amine, C = Carbamate, B = CO_2_, Z = Zwitterion, H^+^ = H_3_O^+^, W = H_2_O;

Some foundations should be laid here: $K_{i}=\frac{k_{-i}}{k_{i}}$

B5 $r_{1}= k_{1}\left[ A \right]C_{\left( * \right)}- k_{-1}{Am}_{(*)}$ $1= \frac{1}{K_{1}}\frac{[A]}{{Am}_{(*)}}C_{(*)}$ if r_1_ = 0.

B6 $r_{2}= k_{2}{Am}_{(*)}p_{CO2}- k_{-2}Z_{(*)}$ $1= K_{2}\frac{Z_{(*)}}{{Am}_{(*)}P_{CO2}}$ if r_2_ = 0.

B7 $r_{3}= k_{3}Z_{(*)}[W]- k_{-3}{Cmate}_{\left( * \right)}[H^{+}]$ $1= K_{3}\frac{{Cmate}_{(*)}[H^{+}]}{Z_{(*)}[W]}=K_{3}'\frac{{Cmate}_{(*)}[H^{+}]}{Z_{(*)}}$ if r_3_ = 0.

B8 $r_{4}= k_{4}{Cmate}_{(*)}- k_{-4}[C]C_{\left( * \right)}$ $1= K_{4}\frac{[C]}{{Cmate}_{(*)}}C_{(*)}$ if r_4_ = 0.

The overall active sites on surface area:

B9 $C_{(*)total}= C_{(*)}+ {Cmate}_{(*)}+ Z_{(*)}+ {Am}_{(*)}$

## 2.1 Rate equations development: B1 to B4

**CASE I:** **B1** is the rate-determine-step (RDS), where the rest equations reached equilibrium:

r = $r_{1}= k_{1}\left[ A \right]C_{\left( * \right)}- k_{-1}{Am}_{(*)}$ ; B1 r_2_ = r_3_ = r_4_ = 0 where B1 is RDS.

Some variables: $\mathrm{Cmate}_{(*)}= K_{4}[C]C_{(*)}$ B10 from eqn B8 where r_4_ = 0.

$Z_{(*)}= K_{3}K_{4}\left[ C \right][H^{+}]C_{(*)}$ B11 from eqn B7*B8 where r_3_ = r_4_ = 0.

${Am}_{(*)}=\frac{K_{2}K_{3}K_{4}\left[ C \right][H^{+}]}{p_{CO2}}C_{(*)}$ B12 from eqn B6*B7*B8 where r_2_=r_3_ = r_4_ = 0.

B9 $C_{(*)total}= C_{(*)}+ {Cmate}_{(*)}+ Z_{(*)}+ {Am}_{(*)}$

= $C_{(*)}+ K_{4}\left[ C \right]C_{\left( * \right)}+ K_{3}K_{4}\left[ C \right]\left[ H^{+} \right]C_{\left( * \right)}+ \frac{K_{2}K_{3}K_{4}\left[ C \right][H^{+}]}{p_{CO2}}C_{(*)}$

= $C_{(*)}\left\{ 1+ K_{4}\left[ C \right]+K_{3}K_{4}\left[ C \right]\left[ H^{+} \right]+ \frac{K_{2}K_{3}K_{4}\left[ C \right][H^{+}]}{p_{CO2}} \right\}$

$C_{(*)}= \frac{C_{(*)total}}{1+ K_{4}\left[ C \right]+K_{3}K_{4}\left[ C \right]\left[ H^{+} \right]+ \frac{K_{2}K_{3}K_{4}\left[ C \right][H^{+}]}{p_{CO2}}}$ B9 for the case where B1 as RDS

Replace B12 into B1

$r_{1}= k_{1}\left[ A \right]C_{\left( * \right)}- k_{-1}{Am}_{(*)}$

$= k_{1}C_{\left( * \right)}\left[ A \right]- k_{-1}K_{2}K_{3}K_{4}\frac{\left[ C \right]\left[ H^{+} \right]}{p_{B}}= k_{1}C_{\left( * \right)}\left\{ \left[ A \right]- K_{1}K_{2}K_{3}K_{4}\frac{\left[ C \right]\left[ H^{+} \right]}{p_{B}} \right\}$

$= k_{1}C_{\left( * \right)}\left\{ \left[ A \right]- K_{A}\frac{\left[ C \right]\left[ H^{+} \right]}{p_{CO2}} \right\}$ where $K_{A}= K_{1}K_{2}K_{3}K_{4}$

Input B9 into B1

$= k_{1}C_{(*)total}\left\{ \frac{\left[ A \right]- K_{A}\frac{\left[ C \right]\left[ H^{+} \right]}{p_{B}}}{1+ K_{4}\left[ C \right]+K_{3}K_{4}\left[ C \right]\left[ H^{+} \right]+ \frac{K_{2}K_{3}K_{4}\left[ C \right][H^{+}]}{p_{B}}} \right\}$ set $K_{B}= k_{1}C_{(*)total}$

$r_{1}= K_{B}\left\{ \frac{\left[ A \right]- K_{A}\frac{\left[ C \right]\left[ H^{+} \right]}{p_{B}}}{1+ K_{4}\left[ C \right]+K_{3}K_{4}\left[ C \right]\left[ H^{+} \right]+ \frac{K_{2}K_{3}K_{4}\left[ C \right][H^{+}]}{p_{B}}} \right\}$ B13

Under absorption conditions, $H^{+}\ll\left[ C \right],\left[ A \right]$, [H^+^] is negligible, the equation can be simplified as

$\boldsymbol{r}_{\boldsymbol{1}}\boldsymbol{=}\boldsymbol{K}_{\boldsymbol{B}}\left\{ \frac{\left[ \boldsymbol{A} \right]}{\boldsymbol{1+}\boldsymbol{K}_{\boldsymbol{4}}\left[ \boldsymbol{C} \right]} \right\}$ B13’ simplifed format of B13

**CASE II:** **B2** is the rate-determine-step (RDS), the rest equations reached equilibrium:

r = $r_{2}= k_{2}{Am}_{(*)}p_{CO2}- k_{-2}Z_{(*)}$ ; B2 r_1_ = r_3_ = r_4_ = 0 where B2 is RDS.

Some variables: ${Cmate}_{(*)}= K_{4}[C]C_{(*)}$ B10 from eqn B8, where r_4_ = 0.

$Z_{(*)}= K_{3}K_{4}\left[ C \right][H^{+}]C_{(*)}$ B11 from eqn B7*B8, where r_3_ = r_4_ = 0.

${Am}_{(*)}=\frac{1}{K_{1}}[A]C_{(*)}$ B14 from eqn B5, where r_1_ = 0.

B9 $C_{(*)total}= C_{(*)}+ {Cmate}_{(*)}+ Z_{(*)}+ {Am}_{(*)}$

= $C_{(*)}+ K_{4}\left[ C \right]C_{\left( * \right)}+ K_{3}K_{4}\left[ C \right]\left[ H^{+} \right]C_{\left( * \right)}+ \frac{\left[ A \right]}{K_{1}}C_{(*)}$

= $C_{(*)}\left\{ 1+ K_{4}\left[ C \right]+K_{3}K_{4}\left[ C \right]\left[ H^{+} \right]+ \frac{\left[ A \right]}{K_{1}} \right\}$

$C_{(*)}= \frac{C_{total}}{1+ K_{4}\left[ C \right]+K_{3}K_{4}\left[ C \right]\left[ H^{+} \right]+ \frac{\left[ A \right]}{K_{1}}}$ B9 for the case where B2 as RDS

Replace B14, B11 into B2

$r_{2}= k_{2}{Am}_{(s)}p_{CO2}- k_{-2}Z_{(*)}$

$=k_{2}\frac{[A]p_{CO2}}{K_{1}}C_{(*)}-k_{-2}K_{3}K_{4}\left[ C \right]\left[ H^{+} \right]C_{\left( * \right)}= k_{2}C_{\left( * \right)}\left\{ \frac{1}{K_{1}}\left[ A \right]p_{B}- K_{2}K_{3}K_{4}\left[ C \right]\left[ H^{+} \right] \right\}$

$= k_{2}C_{\left( * \right)}\left\{ \frac{1}{K_{1}}\left[ A \right]p_{B}- K_{2}K_{3}K_{4}\left[ C \right]\left[ H^{+} \right] \right\}$

Imput B9 into B2

$= k_{2}C_{(*)total}\left\{ \frac{\frac{1}{K_{1}}\left[ A \right]p_{B}- K_{2}K_{3}K_{4}\left[ C \right]\left[ H^{+} \right]}{1+ K_{4}\left[ C \right]+K_{3}K_{4}\left[ C \right]\left[ H^{+} \right]+ \frac{\left[ A \right]}{K_{1}}} \right\}$ where $K_{B}= k_{2}C_{(s)total}$ , $K_{A}= K_{1}K_{2}K_{3}K_{4}$

$r_{2}= K_{B}\left\{ \frac{\left[ A \right]p_{B}- {K_{1}K}_{2}K_{3}K_{4}\left[ C \right]\left[ H^{+} \right]}{K_{1}+ {K_{1}K}_{4}\left[ C \right]+{K_{1}K}_{3}K_{4}\left[ C \right]\left[ H^{+} \right]+[A]} \right\}$

= $K_{B}\left\{ \frac{\left[ A \right]p_{B}- K_{A}\left[ C \right]\left[ H^{+} \right]}{K_{1}+ {K_{1}K}_{4}\left[ C \right]+{K_{1}K}_{3}K_{4}\left[ C \right]\left[ H^{+} \right]+[A]} \right\}$ B15.

Under absorption conditions, $H^{+}\ll\left[ C \right],\left[ A \right]$, H^+^ is negligible, the equation can be simplified as

$\boldsymbol{r}_{\boldsymbol{2}}\boldsymbol{=}\boldsymbol{K}_{\boldsymbol{B}}\left\{ \frac{\frac{\boldsymbol{1}}{\boldsymbol{K}_{\boldsymbol{1}}}\left[ \boldsymbol{A} \right]\boldsymbol{p}_{\boldsymbol{B}}}{\boldsymbol{1+}\boldsymbol{K}_{\boldsymbol{4}}\left[ \boldsymbol{C} \right]\boldsymbol{+}\frac{\left[ \boldsymbol{A} \right]}{\boldsymbol{K}_{\boldsymbol{1}}}} \right\}\boldsymbol{=}\boldsymbol{K}_{\boldsymbol{B}}\left\{ \frac{\left[ \boldsymbol{A} \right]\boldsymbol{p}_{\boldsymbol{B}}}{\boldsymbol{K}_{\boldsymbol{1}}\boldsymbol{+}\boldsymbol{K}_{\boldsymbol{1}}\boldsymbol{K}_{\boldsymbol{4}}\left[ \boldsymbol{C} \right]\boldsymbol{+[A]}} \right\}$ B15’ simplifed format of B15

**CASE III:** **B3** is the rate-determine-step (RDS), where the rest equations reached equilibrium:

r = $r_{3}= k_{3}Z_{(*)}[W]- k_{-3}{Cmate}_{\left( * \right)}[H^{+}]$ ; B3 r_1_ = r_2_ = r_4_ = 0 where B3 is RDS.

Some variables: ${Cmate}_{(*)}= K_{4}[C]C_{(*)}$ B10 from eqn B8, where r_4_ = 0.

$Z_{(*)}= K_{1}K_{2}\left[ A \right]p_{CO2}C_{(*)}$ B16 from eqn B5*B6, where r_1_ = r_2_ = 0.

${Am}_{(*)}=\frac{\left[ A \right]}{K_{1}}C_{(*)}$ B14 from eqn B5, where r_1_ = 0.

B9 $C_{(*)total}= C_{(*)}+ {Cmate}_{(*)}+ Z_{(*)}+ {Am}_{(*)}$

= $C_{(*)}+ K_{4}\left[ C \right]C_{\left( * \right)}+ K_{1}K_{2}\left[ A \right]p_{CO2}C_{\left( * \right)}+ \frac{\left[ A \right]}{K_{1}}C_{(*)}$

= $C_{(*)}\left\{ 1+ K_{4}\left[ C \right]+K_{1}K_{2}\left[ A \right]p_{CO2}+ \frac{\left[ A \right]}{K_{1}} \right\}$

$C_{(*)}= \frac{C_{total}}{1+ K_{4}\left[ C \right]+K_{1}K_{2}\left[ A \right]p_{CO2}+ \frac{\left[ A \right]}{K_{1}}}$ B9 for the case where B3 as RDS

Replace B16, B10 into B3

$r_{3}= k_{3}Z_{(*)}- k_{-3}{Cmate}_{\left( * \right)}[H^{+}]$ [W] is constant move to a new *k_3_*,

$=k_{3}K_{1}K_{2}\left[ A \right]p_{CO2}C_{(*)}-k_{-3}K_{4}[C]\left[ H^{+} \right]C_{\left( * \right)}= k_{3}C_{\left( * \right)}\left\{ K_{1}K_{2}\left[ A \right]p_{B}- K_{3}K_{4}\left[ C \right]\left[ H^{+} \right] \right\}$

$= k_{3}C_{\left( * \right)}\left\{ K_{1}K_{2}\left[ A \right]p_{B}- K_{3}K_{4}\left[ C \right]\left[ H^{+} \right] \right\}$

Imput B9 into B3

$= k_{3}C_{(*)total}\left\{ \frac{K_{1}K_{2}\left[ A \right]p_{B}- K_{3}K_{4}\left[ C \right]\left[ H^{+} \right]}{1+ K_{4}\left[ C \right]+K_{1}K_{2}\left[ A \right]p_{B}+ \frac{\left[ A \right]}{K_{1}}} \right\}$ where $K_{B}= k_{3}C_{(*)total}$ ,

$r_{3}= K_{B}\left\{ \frac{K_{1}K_{2}\left[ A \right]p_{B}- K_{3}K_{4}\left[ C \right]\left[ H^{+} \right]}{1+ K_{4}\left[ C \right]+K_{1}K_{2}\left[ A \right]p_{B}+ \frac{\left[ A \right]}{K_{1}}} \right\}$ = B17.

Under absorption conditions, $H^{+}\ll\left[ C \right],\left[ A \right]$, H^+^ is negligible, the equation can be simplified as

$\boldsymbol{r}_{\boldsymbol{3}}\boldsymbol{=}\boldsymbol{K}_{\boldsymbol{B}}\left\{ \frac{\boldsymbol{K}_{\boldsymbol{1}}\boldsymbol{K}_{\boldsymbol{2}}\left[ \boldsymbol{A} \right]\boldsymbol{p}_{\boldsymbol{B}}}{\boldsymbol{1+}\boldsymbol{K}_{\boldsymbol{4}}\left[ \boldsymbol{C} \right]\boldsymbol{+}\boldsymbol{K}_{\boldsymbol{1}}\boldsymbol{K}_{\boldsymbol{2}}\left[ \boldsymbol{A} \right]\boldsymbol{p}_{\boldsymbol{B}}\boldsymbol{+}\frac{\left[ \boldsymbol{A} \right]}{\boldsymbol{K}_{\boldsymbol{1}}}} \right\}$ B17’ simplifed format of B17

**CASE IV:** **B4** is the rate-determine-step (RDS), where the rest equations reached equilibrium:

r = $r_{4}= k_{4}{Cmate}_{(*)}- k_{-4}[C]C_{\left( * \right)}$ ; B4 r_1_ = r_2_ = r_3_ = 0 where B4 is RDS.

Some variables: ${Cmate}_{(*)}= \frac{K_{1}K_{2}[A]p_{CO2}}{K_{3}[H^{+}]}C_{(*)}$B18 from eqn B5*B6*B7, where r_1_ = r_2_ = r_3_ = 0.

$Z_{(*)}= K_{1}K_{2}\left[ A \right]p_{CO2}C_{(*)}$ B16 from eqn B5*B6, where r_1_ = r_2_ = 0.

${Am}_{(*)}=\frac{\left[ A \right]}{K_{1}}C_{(*)}$ B14 from eqn B5, where r_1_ = 0.

B9 $C_{(*)total}= C_{(*)}+ {Cmate}_{(*)}+ Z_{(*)}+ {Am}_{(*)}$

= $C_{(*)}+\frac{K_{1}K_{2}[A]p_{CO2}}{K_{3}[H^{+}]}C_{(*)}+ K_{1}K_{2}\left[ A \right]p_{CO2}C_{\left( * \right)}+ \frac{\left[ A \right]}{K_{1}}C_{(*)}$

= $C_{(*)}\left\{ 1+ \frac{K_{1}K_{2}[A]p_{CO2}}{K_{3}[H^{+}]}+K_{1}K_{2}\left[ A \right]p_{CO2}+ \frac{\left[ A \right]}{K_{1}} \right\}$

$C_{(*)}= \frac{C_{total}}{1+ \frac{K_{1}K_{2}[A]p_{CO2}}{K_{3}[H^{+}]}+K_{1}K_{2}\left[ A \right]p_{CO2}+ \frac{\left[ A \right]}{K_{1}}}$ B9 for the case where B4 as RDS

Replace B16, B10 into B4

$r_{4}= k_{4}{Cmate}_{(*)}- k_{-4}[C]C_{\left( * \right)}$

$=k_{4}\frac{K_{1}K_{2}[A]p_{CO2}}{K_{3}[H^{+}]}C_{(*)}-k_{-4}[C]C_{\left( * \right)}= k_{4}C_{\left( * \right)}\left\{ \frac{K_{1}K_{2}[A]p_{B}}{K_{3}[H^{+}]}- K_{4}\left[ C \right] \right\}$

$= k_{4}C_{\left( * \right)}\left\{ \frac{K_{1}K_{2}[A]p_{B}}{K_{3}[H^{+}]}- K_{4}\left[ C \right] \right\}$

Input B9 into B4:

$= k_{4}C_{total}\left\{ \frac{\frac{K_{1}K_{2}[A]p_{B}}{K_{3}[H^{+}]}- K_{4}\left[ C \right]}{1+ \frac{K_{1}K_{2}[A]p_{CO2}}{K_{3}[H^{+}]}+K_{1}K_{2}\left[ A \right]p_{B}+ \frac{\left[ A \right]}{K_{1}}} \right\}$ where $K_{B}= k_{4}C_{total}$ ,

$r_{4}= K_{B}\left\{ \frac{\frac{K_{1}K_{2}}{K_{3}}\left[ A \right]p_{B}- K_{3}K_{4}\left[ C \right]\left[ H^{+} \right]}{[H^{+}]+\frac{K_{1}K_{2}\left[ A \right]p_{B}}{K_{3}}+K_{1}K_{2}\left[ A \right][H^{+}]p_{B}+ \frac{\left[ A \right]}{K_{1}}[H^{+}]} \right\}$ = B19.

Under absorption conditions, ${[H}^{+}]\ll\left[ C \right],\left[ A \right]$, H^+^ is negligible, the equation can be simplified as

$\boldsymbol{r}_{\boldsymbol{4}}\boldsymbol{=}\boldsymbol{K}_{\boldsymbol{B}}\left\{ \frac{\frac{\boldsymbol{K}_{\boldsymbol{1}}\boldsymbol{K}_{\boldsymbol{2}}}{\boldsymbol{K}_{\boldsymbol{3}}}\left[ \boldsymbol{A} \right]\boldsymbol{p}_{\boldsymbol{B}}}{\frac{\boldsymbol{K}_{\boldsymbol{1}}\boldsymbol{K}_{\boldsymbol{2}}\left[ \boldsymbol{A} \right]\boldsymbol{p}_{\boldsymbol{B}}}{\boldsymbol{K}_{\boldsymbol{3}}}} \right\}\boldsymbol{=}\boldsymbol{K}_{\boldsymbol{B}}$ B19’ simplifed format of B19, pseudo 0^th^ order

## 2.2 Rate model Validation with experimental datasets of (X_A_, t):

**Case I: The RDS = B1**

**The rate is controlled by amine adsorption onto solid surface**:

$\boldsymbol{r}_{\mathbf{1}}= \boldsymbol{K}_{\boldsymbol{B}}\frac{\left[ \boldsymbol{A} \right]}{\mathbf{1}+ \boldsymbol{k}_{\mathbf{4}}[\boldsymbol{C}]}$

**Sub Case: K_4_[C] << 1:**

$\boldsymbol{r}_{\mathbf{1}}= \boldsymbol{K}_{\boldsymbol{B}}\frac{\left[ \boldsymbol{A} \right]}{\mathbf{1}+ \boldsymbol{k}_{\mathbf{4}}[\boldsymbol{C}]} \approx\boldsymbol{K}_{\boldsymbol{B}}[A]$

$\ln\frac{\boldsymbol{1}}{\boldsymbol{1-}\boldsymbol{X}_{\boldsymbol{A}}}\mathbf{=}\boldsymbol{K}_{\boldsymbol{B}}\boldsymbol{t}$

**Sub Case: K_4_[C] >> 1:**

$\boldsymbol{r}_{\mathbf{1}}= \boldsymbol{K}_{\boldsymbol{B}}\frac{\left[ \boldsymbol{A} \right]}{\mathbf{1}+ \boldsymbol{k}_{\mathbf{4}}[\boldsymbol{C}]} \approx\boldsymbol{K}_{\boldsymbol{B}}\frac{\left[ \boldsymbol{A} \right]}{\boldsymbol{k}_{\mathbf{4}}\left[ \boldsymbol{C} \right]}$

$\ln\frac{\boldsymbol{1}}{\boldsymbol{1-}\boldsymbol{X}_{\boldsymbol{A}}}\mathbf{-}\boldsymbol{X}_{\boldsymbol{A}}\mathbf{=}\boldsymbol{K}^{\boldsymbol{'}}\boldsymbol{t}$ $\boldsymbol{K}^{\boldsymbol{'}}\boldsymbol{=}\frac{\boldsymbol{2}\boldsymbol{K}_{\boldsymbol{B}}}{\boldsymbol{K}_{\boldsymbol{4}}\boldsymbol{C}_{\boldsymbol{A}\boldsymbol{0}}}$

**CASE II: The RDS = B2, the rate is controlled by N-C bond formation of carbamate formation**:

$\boldsymbol{r}_{\boldsymbol{2}}\boldsymbol{=}\boldsymbol{K}_{\boldsymbol{B}}\left\{ \frac{\left[ \boldsymbol{A} \right]\boldsymbol{p}_{\boldsymbol{B}}}{\boldsymbol{K}_{\boldsymbol{1}}\boldsymbol{+}\boldsymbol{K}_{\boldsymbol{1}}\boldsymbol{K}_{\boldsymbol{4}}\left[ \boldsymbol{C} \right]\boldsymbol{+[A]}} \right\}\boldsymbol{\approx}\boldsymbol{K}_{\boldsymbol{B}}\left\{ \frac{\left[ \boldsymbol{A} \right]\boldsymbol{p}_{\boldsymbol{B}}}{\boldsymbol{K}_{\boldsymbol{1}}\boldsymbol{K}_{\boldsymbol{4}}\left[ \boldsymbol{C} \right]\boldsymbol{+[A]}} \right\}$

$\boldsymbol{k}_{\boldsymbol{a}}\left( \ln\frac{\boldsymbol{1}}{\boldsymbol{1-}\boldsymbol{X}_{\boldsymbol{A}}}\mathbf{-}\boldsymbol{X}_{\boldsymbol{A}} \right)\mathbf{+}\boldsymbol{X}_{\boldsymbol{A}}\mathbf{=}\boldsymbol{K}^{\boldsymbol{''}}\boldsymbol{t}$

***ka* = 0.5 K_1_ K_4_ ;** $K^{''}=\frac{\boldsymbol{K}_{\boldsymbol{B}}p_{B}}{C_{A0}}$

**Sub Case: *ka* = 0.05; *ka* = 0.01; *ka* = 0.005;**

**Special: *ka* = 0;** $\boldsymbol{X}_{\boldsymbol{A}}\mathbf{=}\boldsymbol{K}^{\boldsymbol{''}}\boldsymbol{t}$
