# Supplementary material for: Eley–Rideal model of heterogeneous catalytic carbamate formation based on CO2–MEA absorptions with CaCO3, MgCO3 and BaCO3
Source: R Soc Open Sci. 2019 May 1;6(5):190311. doi: 10.1098/rsos.190311 (PMC6549977; doi:10.1098/rsos.190311)
Supplement: Supplementary materials B the experimental data [file rsos190311supp2.docx]

**Support Information B:**

1. The experimental results of CO_2_ MEA absorption profiles under non-catalytic and catalytic conditions, which is the data for Fig 3-5.

Table SA.1 CO_2_-MEA with 0-20g CaCO_3_

| MEA Conc | CO_2_ loading α | X_A_ ^a^ | Carbamate | MEA | time(s) of different mass of Catalyst CaCO_3_ | | | | | |
| --- | --- | --- | --- | --- | --- | --- | --- | --- | --- | --- |
| 1.0 M | mol/mol |  | mol/L | mol/L | 0g | 5g | 10g | 15g | 20g | 25g |
|  | 0.00 | 0.0 | 0.00 | 1.00 | 0 | 0 | 0 | 0 | **0** |  |
|  | 0.20 | 0.40 | 0.20 | 0.60 | 110 | 110 | 125 | 95 | **100** |  |
| CO_2_ flowrate | 0.25 | 0.50 | 0.25 | 0.50 | 145 | 140 | 140 | 135 | **130** |  |
| 1.0 L / min | 0.30 | 0.60 | 0.30 | 0.40 | 195 | 185 | 210 | 180 | **175** |  |
|  | 0.35 | 0.70 | 0.35 | 0.30 | 275 | 235 | 260 | 245 | **200** |  |
|  | 0.40 | 0.80 | 0.40 | 0.20 | 430 | 375 | 325 | 315 | **260** |  |
|  | 0.50 | 1.00 | > 0.40 | 0.00 | 820 | 600 | 515 | 480 | **420** |  |
|  |  |  |  |  |  |  |  |  |  |  |
|  | CO_2_ loading | X_A_ | Carbamate | MEA | t(s) of different mass of Catalyst CaCO_3_ | | | | | |
| 3.0 M | mol/mol | % | mol/L | mol/L | 0g | 5g | 10g | 15g | 20g | 25g |
|  | 0.00 | 0.0 | 0.00 | 3.00 | 0 | 0 | 0 | 0 | **0** | 0 |
|  | 0.20 | 0.40 | 0.60 | 1.80 | 245 | 270 | 255 | 215 | **230** | 250 |
| CO_2_ flowrate | 0.25 | 0.50 | 0.75 | 1.50 | 335 | 350 | 325 | 285 | **305** | 335 |
| 1.0 L / min | 0.30 | 0.60 | 0.90 | 1.20 | 440 | 460 | 425 | 370 | **385** | 425 |
|  | 0.35 | 0.70 | 1.05 | 0.90 | 590 | 580 | 545 | 480 | **480** | 530 |
|  | 0.40 | 0.80 | 1.20 | 0.60 | 830 | 770 | 710 | 640 | **625** | 695 |
|  | 0.50 | 1.00 | > 1.2 | 0.00 | 2280 | 1895 | 1740 | 1525 | **1395** | 1685 |
|  |  |  |  |  |  |  |  |  |  |  |
|  | CO_2_ loading | X_A_ | Carbamate | MEA | t(s) of different mass of Catalyst CaCO_3_ | | | | | |
| 5.0 M | mol/mol | % | mol/L | mol/L | 0g | 5g | 10g | 15g | 20g | 25g |
|  | 0.00 | 0.0 | 0.00 | 5.00 | 0 | 0 | 0 | 0 | **0** | 0 |
|  | 0.20 | 0.40 | 1.00 | 3.00 | 535 | 350 | 340 | 325 | **275** | 335 |
| CO_2_ flowrate | 0.25 | 0.50 | 1.25 | 2.50 | 645 | 465 | 435 | 430 | **360** | 430 |
| 1.5 L / min | 0.30 | 0.60 | 1.50 | 2.00 | 865 | 600 | 570 | 550 | **460** | 570 |
|  | 0.35 | 0.70 | 1.75 | 1.50 | 1125 | 765 | 705 | 700 | **580** | 750 |
|  | 0.40 | 0.80 | 2.00 | 1.00 | 1500 | 1020 | 910 | 895 | **735** | 960 |
|  | 0.50 | 1.00 | > 2.0 | 0.00 | 2820 | 2340 | 2195 | 2100 | **1850** | 2235 |
|  |  |  |  |  |  |  |  |  |  |  |

1. The conversion was calculated with X_A_ = 1 - 2 α (α < 0.40; X_A_ < 0.80)

Table SA.2 CO_2_-MEA with 0-20g MgCO_3_.

| MEA Conc | CO_2_ loading | X_A_ | Carbamate | MEA | t(s) of differnet mass of Catalyst MgCO_3_ | | | | |
| --- | --- | --- | --- | --- | --- | --- | --- | --- | --- |
| 1.0 M | mol/mol |  | mol/L | mol/L | 0g | 5g | 10g | 15g | 20g |
|  | 0.00 | 0.0 | 0.00 | 1.00 | 0 | 0 | 0 | **0** | 0 |
|  | 0.20 | 0.40 | 0.20 | 0.60 | 110 | 90 | 90 | **80** | 90 |
| CO_2_ flowrate | 0.25 | 0.50 | 0.25 | 0.50 | 145 | 115 | 120 | **120** | 120 |
| 1.0 L / min | 0.30 | 0.60 | 0.30 | 0.40 | 195 | 150 | 150 | **145** | 150 |
|  | 0.35 | 0.70 | 0.35 | 0.30 | 275 | 190 | 190 | **185** | 195 |
|  | 0.40 | 0.80 | 0.40 | 0.20 | 430 | 250 | 245 | **230** | 245 |
|  | 0.50 | 1.00 | > 0.40 | 0.00 | 720 | 540 | 510 | **420** | 480 |
|  |  |  |  |  |  |  |  |  |  |
|  |  |  |  |  |  |  |  |  |  |
| Concentration | CO_2_ loading | X_A_ | Carbamate | MEA | t(s) of differnet mass of Catalyst MgCO_3_ | | | | |
| 3.0 M | mol/mol |  | mol/L | mol/L | 0g | 5g | 10g | 15g | 20g |
|  | 0.00 | 0.0 | 0.00 | 3.00 | 0 | 0 | 0 | 0 | **0** |
|  | 0.20 | 0.40 | 0.60 | 1.80 | 245 | 280 | 265 | 265 | **255** |
| CO_2_ flowrate | 0.25 | 0.50 | 0.75 | 1.50 | 335 | 345 | 340 | 345 | **335** |
| 1.0 L / min | 0.30 | 0.60 | 0.90 | 1.20 | 440 | 450 | 445 | 445 | **435** |
|  | 0.35 | 0.70 | 1.05 | 0.90 | 590 | 585 | 565 | 560 | **550** |
|  | 0.40 | 0.80 | 1.20 | 0.60 | 830 | 790 | 745 | 730 | **715** |
|  | 0.50 | 1.00 | > 1.2 | 0.00 | 2280 | 1890 | 1560 | 1525 | **1450** |
|  |  |  |  |  |  |  |  |  |  |
|  |  |  |  |  |  |  |  |  |  |
| Concentration | CO_2_ loading | X_A_ | Carbamate | MEA | t(s) of differnet mass of Catalyst MgCO_3_ | | | | |
| 5.0 M | mol/mol |  | mol/L | mol/L | 0g | 5g | 10g | 15g | 20g |
|  | 0.00 | 0.0 | 0.00 | 5.00 | 0 | 0 | 0 | **0** | 0 |
|  | 0.20 | 0.40 | 1.00 | 3.00 | 535 | 350 | 345 | **340** | 355 |
| CO_2_ flowrate | 0.25 | 0.50 | 1.25 | 2.50 | 645 | 460 | 455 | **430** | 455 |
| 1.5 L / min | 0.30 | 0.60 | 1.50 | 2.00 | 865 | 625 | 595 | **585** | 610 |
|  | 0.35 | 0.70 | 1.75 | 1.50 | 1125 | 790 | 765 | **745** | 750 |
|  | 0.40 | 0.80 | 2.00 | 1.00 | 1500 | 1060 | 1010 | **995** | 1010 |
|  | 0.50 | 1.00 | > 2.0 | 0.00 | 2820 | 2640 | 2425 | **2280** | 2320 |
|  |  |  |  |  |  |  |  |  |  |

Table SA.3 CO_2_-MEA with 0-25g BaCO_3_.

| MEA Conc | CO_2_ loading | X_A_ | Carbamate | MEA | t(s) of differnet mass of Catalyst BaCO_3_ | | | | | |
| --- | --- | --- | --- | --- | --- | --- | --- | --- | --- | --- |
| 1.0 M | mol/mol |  | mol/L | mol/L | 0g | 5g | 10g | 15g | 20g | 25g |
|  | 0.00 | 0.0 | 0.00 | 1.00 | 0 | 0 | 0 | 0 | 0 | **0** |
|  | 0.20 | 0.40 | 0.20 | 0.60 | 110 | 90 | 125 | 115 | 105 | **90** |
| CO_2_ flowrate | 0.25 | 0.50 | 0.25 | 0.50 | 145 | 120 | 165 | 145 | 135 | **120** |
| 1.0 L / min | 0.30 | 0.60 | 0.30 | 0.40 | 195 | 170 | 210 | 195 | 180 | **155** |
|  | 0.35 | 0.70 | 0.35 | 0.30 | 275 | 225 | 265 | 245 | 240 | **190** |
|  | 0.40 | 0.80 | 0.40 | 0.20 | 430 | 350 | 345 | 325 | 300 | **240** |
|  | 0.50 | 1.00 | > 0.40 | 0.00 | 820 | 710 | 720 | 625 | 590 | **455** |
|  |  |  |  |  |  |  |  |  |  |  |
|  | loading | X_A_ | Carbamate | MEA | t(s) of differnet mass of Catalyst BaCO_3_ | | | | | |
| 3.0 M | mol/mol |  | mol/L | mol/L | 0g | 5g | 10g | 15g | 20g | 25g |
|  | 0.00 | 0.0 | 0.00 | 3.00 | 0 | 0 | 0 | 0 | 0 | **0** |
|  | 0.20 | 0.40 | 0.60 | 1.80 | 245 | 260 | 265 | 255 | 265 | **240** |
| CO_2_ flowrate | 0.25 | 0.50 | 0.75 | 1.50 | 335 | 340 | 340 | 335 | 340 | **320** |
| 1.0 L / min | 0.30 | 0.60 | 0.90 | 1.20 | 440 | 455 | 460 | 440 | 450 | **415** |
|  | 0.35 | 0.70 | 1.05 | 0.90 | 590 | 585 | 580 | 565 | 560 | **525** |
|  | 0.40 | 0.80 | 1.20 | 0.60 | 830 | 770 | 765 | 735 | 720 | **670** |
|  | 0.50 | 1.00 | > 1.2 | 0.00 | 2280 | 1920 | 1650 | 1575 | 1560 | **1425** |
|  |  |  |  |  |  |  |  |  |  |  |
|  | loading | X_A_ | Carbamate | MEA | t(s) of differnet mass of Catalyst BaCO_3_ | | | | | |
| 5.0 M | mol/mol |  | mol/L | mol/L | 0g | 5g | 10g | 15g | 20g | 25g |
|  | 0.00 | 0.0 | 0.00 | 5.00 | 0 | 0 | 0 | 0 | 0 | **0** |
|  | 0.20 | 0.40 | 1.00 | 3.00 | 535 | 430 | 340 | 360 | 370 | **335** |
| CO_2_ flowrate | 0.25 | 0.50 | 1.25 | 2.50 | 645 | 565 | 450 | 480 | 470 | **445** |
| 1.5 L / min | 0.30 | 0.60 | 1.50 | 2.00 | 865 | 760 | 595 | 615 | 600 | **575** |
|  | 0.35 | 0.70 | 1.75 | 1.50 | 1125 | 985 | 745 | 800 | 750 | **735** |
|  | 0.40 | 0.80 | 2.00 | 1.00 | 1500 | 1300 | 1085 | 1045 | 985 | **955** |
|  | 0.50 | 1.00 | > 2.0 | 0.00 | 2820 | 2410 | 2235 | 2185 | 2065 | **2060** |
|  |  |  |  |  |  |  |  |  |  |  |

1. The ratio of slopes of k_B_ [CO_2_] / kz [CO_2_] of rate equation: $\ln\frac{1}{1-X_{A}}=k t$.

The slopes represented the enhancement of catalysis for catalytic absorption vs non-catalytic absorption.

Table SB.1 The catalysis of CaCO_3_, MgCO_3_, and BaCO_3_ compare to kz.

| CaCO_3_ | non catalytic  as 1.0 | **φ** = k_B_ [CO_2_] / kz [CO_2_] ^a^ | | | | |
| --- | --- | --- | --- | --- | --- | --- |
| m (g) | 0 | 5 | 10 | 15 | **20** | 25 |
| 1.0 M | 1.00 | 1.19 | 1.32 | 1.35 | **1.68** |  |
| 3.0 M | 1.00 | 1.11 | 1.21 | 1.37 | **1.32** | 1.21 |
| 5.0 M | 1.00 | 1.47 | 1.64 | 1.66 | **2.02** | 1.54 |
|  |  |  |  |  |  |  |
| MgCO_3_ | non catalytic | **φ** = k_B_ [CO_2_] / kz [CO_2_] | | | | |
| m (g) | 0 | 5 | 10 | **15** | 20 | 25 |
| 1.0 M | 1.00 | 1.76 | 1.78 | **1.86** | 1.76 |  |
| 3.0 M | 1.00 | 1.11 | 1.16 | **1.16** | 1.21 |  |
| 5.0 M | 1.00 | 1.41 | 1.48 | **1.50** | 1.49 |  |
|  |  |  |  |  |  |  |
| BaCO_3_ | non catalytic | **φ** = k_B_ [CO_2_] / kz [CO_2_] | | | | |
| m (g) | 0 | 5 | 10 | 15 | 20 | **25** |
| 1.0 M | 1.00 | 1.24 | 1.27 | 1.35 | 1.43 | **1.81** |
| 3.0 M | 1.00 | 1.11 | 1.11 | 1.16 | 1.16 | **1.26** |
| 5.0 M | 1.00 | 1.11 | 1.39 | 1.39 | 1.57 | **1.57** |
|  |  |  |  |  |  |  |
